# PEGylated liposomal fluopsin C triggers cuproptosis and ferroptosis pathways and suppresses 3D tumor spheroid growth in NCI-H460 cells

**DOI:** 10.1007/s00204-026-04315-0

**Published:** 2026-02-27

**Authors:** Luan Vitor Alves de Lima, Matheus Felipe da Silva, Liana Martins de Oliveira, Maria Claudia Terkelli de Assis, Isabella Cristina Oliveira Carvalho, Isaura Maria Fuzinatto, Simone Cristine Semprebon, Renan Vinícius de Oliveira Nocetti, Danielle Lazarin-Bidoia, Celso Vataru Nakamura, Ingrid Felicidade, Sandra Regina Lepri, Phelipe Oliveira Favaron, Mickely Liuti Dealis, Luis Fernando Cabeça, Galdino Andrade Filho, Mário Sérgio Mantovani

**Affiliations:** 1 Laboratory of Toxicological Genetics, Department of General Biology, Center for Biological Sciences, State University of Londrina, Londrina, Paraná Brazil; 2 Laboratory of Technological Innovation in Drug and Cosmetic Development, Department of Basic Health Sciences, Maringá State University, Maringá, Paraná Brazil; 3 Laboratory of Microbial Ecology, Department of Microbiology, Center for Biological Sciences, State University of Londrina, Londrina, Paraná Brazil; 4 Chemistry Laboratory, Federal Technological University of Paraná, Londrina, Paraná Brazil

**Keywords:** Metallodrug, Natural products, Non-apoptotic cell death, Regulated cell death, Targeted drug delivery

## Abstract

Copper-based metallodrugs are promising anticancer agents, but their toxicity limits clinical translation. This study evaluated the toxicological and molecular effects of PEGylated Fluopsin C (PEG-FlpC) in in vitro human non-small-cell lung carcinoma (NCI-H460) cells cultured as 2D monolayer and 3D spheroids. Cells were exposed to PEG-FlpC at its IC_50_ (1.0 µM) for 24 h. Flow cytometry revealed G₁-phase arrest and non-apoptotic cell death. PEG-FlpC markedly suppressed spheroid growth, with 2 × IC_50_-treated spheroids growing only 58.1% compared to 131.6% in the control. Ultrastructural analyses showed mitochondrial clustering and swelling, ER dilation, and cytoplasmic vacuolization. Gene profiling demonstrated downregulation of *TP53* and *BECN1*, with upregulation of *TP73* and *SQSTM1*, consistent with autophagy blockade and stress activation. PEG-FlpC modulated ferroptosis-related (*GPX4*, *SLC7A11*, and *TFRC*) and cuproptosis-related (*ATP7B*, *MTF1*, *DLAT*, and *CDKN2A*) genes. Pharmacological inhibition confirmed a predominantly copper-mediated mechanism, as TTM (10 µM) restored 77.7% of metabolic activity. Combined inhibition with TTM and Fer-1 (1 µM) further increased viability to 89.4%, indicating that PEG-FlpC induces non-apoptotic regulated cell death through an interconnected cuproptosis–ferroptosis mechanism associated with mitochondrial dysfunction.

**Supplementary Information:**

The online version contains supplementary material available at 10.1007/s00204-026-04315-0.

## Introduction

Non-small cell lung cancer (NSCLC) remains one of the most challenging malignancies, with survival limited by intrinsic and acquired resistance to current therapies (Ashrafi et al. 2022). This resistance is largely driven by apoptosis evasion linked to *TP53* mutations, highlighting the need for strategies that activate alternative regulated cell death (RCD) pathways (Vasan et al. 2019). Copper-based metallodrugs have emerged as promising candidates since they interact with multiple molecular targets, disrupt redox homeostasis, and trigger non-apoptotic cell death (Guo et al. 2023).

Among these agents, Fluopsin C (FlpC), a natural copper complex (Otsuka et al. 1972), exhibits potent antiproliferative effects via oxidative stress, DNA damage, and mitochondrial dysfunction (Ma et al. 2013; Alves de Lima et al. 2022). However, its free form shows toxicity that limits translational use (Navarro et al. 2019). Nanotechnology offers a rational approach to enhance biocompatibility and pharmacokinetics. PEGylated liposomes improve solubility, stability, and tumor accumulation through the enhanced permeability and retention effect (Fulton and Najahi-Missaoui 2023), reducing copper-related toxicity while maintaining activity (Dealis Gomes et al. 2025).

Based on this premise, Dealis Gomes et al. (2025) developed PEGylated FlpC with high encapsulation efficiency, physicochemical stability, and absence of lethality up to 8 mg/kg in vivo, confirming improved systemic safety. These findings support PEG-FlpC as a biocompatible model to study copper-induced antiproliferative effects. Understanding how PEGylation modifies copper-dependent responses is crucial to define the balance between therapeutic efficacy and metal-induced stress.

At the molecular level, metal complexes can activate unconventional RCD pathways dependent on intracellular metal homeostasis. Cuproptosis, a copper-driven process marked by aggregation of lipoylated mitochondrial enzymes (Tsvetkov et al. 2022), and ferroptosis, an iron-dependent lipid peroxidation pathway (Dixon et al. 2012), represent distinct yet interconnected death mechanisms (Liu and Chen 2024). Identifying whether PEG-FlpC modulates these processes is key to elucidating its toxicodynamic profile.

To further explore these mechanisms under tumor-like conditions, three-dimensional (3D) spheroids provide a physiologically relevant model to evaluate cytotoxicity and cell death (Nunes et al. 2019; Han et al. 2021). Thus, this study investigates whether PEG-FlpC enhances cytotoxic efficacy by co-activating ferroptotic and cuproptotic pathways in human NSCLC cells. Integrating 2D and 3D systems with ultrastructural, flow cytometric, and transcriptional analyses, we clarify how PEG-FlpC induces organelle stress and non-apoptotic RCD, offering mechanistic insight into copper-induced oxidative and proteotoxic stress and supporting the development of safer copper-based nanotherapeutics.

## Materials and methods

### Cell culture and treatment

The human NSCLC cell line NCI-H460 (RRID: CVCL_0459) was obtained from the State University of Campinas (UNICAMP, São Paulo, Brazil), with mycoplasma-free status confirmed by RT-qPCR. Cells were cultured in MEM (Gibco^®^, Cat. 41500-034) supplemented with 10% fetal bovine serum (FBS; Gibco^®^, Cat. 12657-029), 1 mM sodium pyruvate, and 1% antibiotic–antimycotic (10,000 U/mL penicillin, 10,000 µg/mL streptomycin, and 250 µg/mL amphotericin B) at 37 °C and 5% CO₂. Cell viability (> 90%) was verified by trypan blue exclusion using the Countess II FL automated cell counter (Life Technologies^®^).

FlpC (C_4_H_8_CuN_2_O_2_S_2_; MW: 243.8 g/mol) was provided by the Microbial Ecology Laboratory, State University of Londrina. The PEGylated liposomal formulation (PEG-FlpC; DSPE-PEG + FlpC) was supplied by the Federal Technological University of Paraná (UTFPR, Londrina, Brazil) and prepared as described by Dealis Gomes et al. (2025). The lyophilized formulation was stored at 4 °C and resuspended in complete medium immediately before use. Empty PEGylated liposomes (vehicle control for PEG-FlpC) were prepared under identical conditions without drug incorporation. Their biological inertness was experimentally validated, as empty liposomes did not significantly affect cell viability compared to the negative control, consisting of untreated cells (Supplementary Table S1). A 24.3 mM stock solution of free FlpC (F-FlpC) was prepared in 100% DMSO. Doxorubicin (DXR; 8 µM; Pharmacia, CAS 23214-92-8) and camptothecin (CPT; 10 µM; Sigma–Aldrich, Cat. C9911) served as positive controls, with concentrations selected based on a previously published study using NCI-H460 cells (da Silva et al. 2023). The IC_50_ of PEG-FlpC was used as the reference concentration, corresponding to a 50% reduction in metabolic activity after 24 h. This condition was suitable to investigate regulated cell death pathways without complete loss of viability. F-FlpC was tested at the equivalent concentration to enable direct comparison between formulations. For 3D spheroids, the ½× and 2× IC_50_ concentrations were also included to assess dose–response relationships and threshold effects.

### Monolayer assays

#### Cell metabolic activity (Resazurin assay)

NCI-H460 cells (1 × 10^4^/well) were seeded in 96-well plates, stabilized for 24 h, and treated with F-FlpC, PEG-FlpC (0.4–2 µM), vehicle, or DXR for 24 h. Resazurin (60 µM) was added for 2 h, and fluorescence was read (excitation 520 nm, emission 580–640 nm) using a Glomax^®^ spectrophotometer. Viability was expressed as fluorescence of treatment/negative control × 100. IC_12,5_, IC_25_, and IC_50_ were calculated by nonlinear regression using GraphPad Prism 10.1.2. Data represent mean ± SD from three biological replicates.

#### Morphological analyses by time-lapse and electron microscopy

NCI-H460 cells (2.5 × 10^4^/well) were seeded in 48-well plates and treated with PEG-FlpC (IC₅₀) or empty liposomes for 24 h. Time-lapse images were acquired every 3 min using the EVOS^®^ FL Auto system (Thermo Fisher Scientific, USA). For transmission electron microscopy (TEM), cells grown on round glass coverslips in 24-well plates were treated with PEG-FlpC (IC_50_) for 24 h, washed with PBS, fixed in 2.5% glutaraldehyde in 0.1 M sodium cacodylate buffer (pH 7.2) for 24 h, and post-fixed in 1% osmium tetroxide, 0.8% potassium ferrocyanide, and 10 mM CaCl_2_ in the same buffer for 1 h. Samples were dehydrated in graded acetone (30–100%), embedded in EPON™ epoxy resin (60 °C, 72 h), sectioned (60–70 nm), stained with uranyl acetate and lead citrate, and examined using a JEOL JEM-1400 microscope. For scanning electron microscopy (SEM), cells were fixed and post-fixed under the same conditions, dehydrated in graded ethanol (30–100%), critical-point dried, gold-coated, and examined using a FEI Quanta 250 microscope. All electron microscopy analyses (SEM and TEM) were performed in independent biological replicates. For each experimental condition, at least 20 randomly selected microscopic fields per sample, corresponding to approximately 50 cells, were evaluated. Fields with preparation artifacts or poor preservation were excluded.

#### Flow cytometry analysis (cell cycle and Annexin V/7-AAD)

Cells (3.2 × 10^5^/well) were seeded in 6-well plates, stabilized for 24 h, and treated for 24 h with F-FlpC, PEG-FlpC (IC_12,5_, IC_25_ e IC_50_), empty liposomes, or CPT. For cell cycle analysis, cells were collected, washed, trypsinized, centrifuged (644 × g, 5 min), resuspended in citrate/Triton buffer, and stained with propidium iodide (50 µg/mL). A total of 10,000 events per sample were acquired using a Guava^®^ EasyCyte flow cytometer (Merck Millipore). For cell death analysis, apoptosis and non-apoptotic death were evaluated using the Guava^®^ Nexin Kit (Merck Millipore, Cat. 4500 − 0450) with Annexin V and 7-AAD staining, and data were analyzed in FCS Express™ 7 software (De Novo Software). Based on the absence of apoptotic morphology observed by morphological analysis and time-lapse imaging, and supported by subsequent evidence of ferroptosis and cuproptosis activation, the Annexin V⁺/7-AAD⁺ population was classified as non-apoptotic cell death rather than late apoptosis. Data represent mean ± SD from three biological replicates.

#### Mitochondrial function and plasma membrane assessment by fluorescence microscopy

After 24 h of treatment with PEG-FlpC or empty liposomes, cells were stained with Hoechst 33,342 combined with Rhodamine 123 (5 µg/mL) to assess mitochondrial membrane potential, or with fluorescein diacetate (FDA) and propidium iodide (PI; 50 µg/mL) to evaluate plasma membrane integrity and viability. After 30 min of incubation, fluorescence images were acquired at 200× magnification using the EVOS^®^ FL Auto system.

### Three-dimensional tumor spheroid model

#### Spheroid generation and pre-selection

Spheroids were generated by the liquid overlay method. Plates (96-well) were precoated with 50 µL of 1.5% low-melting-point agarose in serum-free medium (Friedrich et al. 2009). Cells (3 × 10^3^/well) were seeded in 150 µL of complete medium, centrifuged (500 × g, 2 min), and incubated for 3 days to allow spheroidization. Treatments were applied by adding 50 µL of fresh medium containing compounds, resulting in a final volume of 200 µL per well. Uniform spheroids were pre-selected for treatment. For single-cell assays, spheroids were dissociated with Accutase^®^ (150 µL, 37 °C, 10 min, 1400 rpm) (Grässer et al. 2018).

#### Growth kinetics, proliferative recovery, and mitochondrial integrity

Spheroids (*n* = 5/condition) were treated with PEG-FlpC (½×, 1×, 2× IC_50_), F-FlpC, empty liposomes, or DXR, and bright-field images were captured at 0, 24, 48, and 72 h using the EVOS™ FL Auto microscope. Spheroid volumes (mm³) were calculated from AnaSP/ReViSP binary masks (Piccinini 2015; Piccinini et al. 2015). After 72 h, spheroids were transferred to adherent plates for 48 h to evaluate proliferative recovery. For mitochondrial and membrane integrity assessment, spheroids treated for 72 h with PEG-FlpC or empty liposomes were stained for mitochondrial function and membrane integrity as described above for monolayer culture. Data represent mean ± SD from three independent replicates.

#### DNA damage assessment by comet assay

DNA damage was evaluated by alkaline comet assay (Collins 2004). Spheroids (*n* = 10/condition) were treated with PEG-FlpC (½×, 1×, 2× IC_50_) for 3 h, dissociated, centrifuged, and resuspended in PBS. Cell preparation, slide preparation, electrophoresis, and staining were performed according to Alves de Lima et al. (2022). Slides were stained with ethidium bromide (0.002 µg/mL), and 100 comets per treatment were analyzed using the EVOS^®^ FL Auto Cell Imaging System (Thermo Fisher Scientific, USA) at 200× magnification. Comets were visually scored from 0 (no damage) to 4 (maximal damage). The DNA damage index was calculated as: DNA damage index = (0 × n_₀_) + (1 × n_₁_) + (2 × n_₂_) + (3 × n_₃_) + (4 × n_₄_), where *ni* is the number of comets in each class (Collins 2004). Data represent mean ± SD (*n* = 3).

#### Cell cycle and cell death analysis in spheroids

Spheroids were treated for 24 h with F-FlpC, PEG-FlpC (½ IC_50_, IC_50_, 2×IC_50_), empty liposome, or CPT. After treatment, ten spheroids per condition were collected and dissociated. The resulting single-cell suspensions were stained for cell cycle and cell death analysis as described in section for 2D cultures. Data represent mean ± SD (*n* = 3).

#### Relative quantification of poly-A mRNA by RT-qPCR

Poly-A mRNA was quantified from 20 spheroids per treatment (PEG-FlpC IC_50_ or empty liposome). Total RNA was extracted (RNeasy^®^ Mini Kit, Qiagen), quantified (A260/A280 = 1.9–2.0), and reverse-transcribed (500 ng RNA), as described by Zanetti et al. (2019). Reactions contained 5 µL PowerUP™ SYBR Green Master Mix (Thermo Fisher Scientific, Cat. 01197264), primers at a final concentration of 0.45 µM each (corresponding to 0.5 µL of a 10 µM working solution), and 5 µL of diluted cDNA (1:10). Amplification was performed for 45 cycles on a CFX96 Touch™ Real-Time PCR Detection System (Bio-Rad Laboratories, USA) following the manufacturer’s recommendations for the PowerUP™ SYBR Green Master Mix. Melting curves confirmed specificity (55–95 °C, ramp rate 0.5 °C/s). Targets included genes involved in cell cycle (*TP53*, *TP73*, *C-MYC*, *CDKN1A*, and *BIRC5*), cuproptosis (*FDX1*, *SLC31A1*, *MTF1*, *DLAT*, *LIPT1*, *ATP7B*, *BBC3*, *CDKN2A*, and *SOD1*), ferroptosis (*GPX4*, *GPX1*, *SLC7A11*, *SLC3A2*, *ACSL4*, *NFE2L2*, *NQO1*, *ATF4*, *TFRC*, *GCLM*, and *GSR*), necroptosis (*RIPK1*, *RIPK3*, and *MLKL*), autophagy (*SQSTM1*, *BECN1*, and *MTOR*), endoplasmic reticulum stress (*ERN1* and *HSPA5*), and DNA damage (*GADD45A* and *PARP1*). Primer sequences are listed in Supplementary Table 2. Data are presented as mean ± SD of fold change (*n* = 3).

### Functional and mechanistic assays of cuproptosis and ferroptosis

#### Pharmacological modulation

To elucidate the involvement of cuproptosis and/or ferroptosis in the cytotoxic effects of FlpC, NCI-H460 cells were exposed to specific pharmacological modulators. Ferroptosis and cuproptosis were inhibited by Ferrostatin-1 (Fer-1; Sigma-Aldrich, Cat. SML0583; 1 µM) and ammonium tetrathiomolybdate (TTM; Sigma-Aldrich, Cat. 323446-1G; 10 µM), respectively. Cells were pretreated with each inhibitor for 1 h before exposure to PEG-FlpC (IC₅₀) or vehicle control for 24 h. For positive controls, cells were treated with the ferroptosis inducer ML162 (TargetMol, Cat. T8970; 5 µM) or with the cuproptosis inducer Elesclomol (TargetMol, Cat. T6170; 100 nM) in combination with CuSO₄·5 H₂O (1 µM; referred to as ES–Cu) for 24 h under the same experimental conditions. Following pharmacological modulation, cell metabolic activity was evaluated using the resazurin assay, performed as described above for monolayer culture. Metabolic activity was expressed relative to untreated controls (set as 100%). Data represent mean ± SD (*n* = 3).

#### Mitochondrial membrane potential (Δψmit)

Mitochondrial membrane potential (Δψmit) was evaluated by Rhodamine 123 retention. NCI-H460 cells (1.18 × 10^5^/well) were seeded in 12-well plates, stabilized for 24 h, and pretreated for 1 h with Fer-1 or TTM or Fer-1 + TTM before exposure to PEG-FlpC (IC₅₀) or empty liposomes for 24 h. After treatment, cells were detached, centrifuged, and incubated with Rhodamine 123 (1 µg/mL, 30 min, 37 °C). Fluorescence was acquired using a Guava^®^ EasyCyte flow cytometer (Merck Millipore; 5,000 events/sample) and analyzed in FCS Express™ 7 software (De Novo Software). Results were expressed as the percentage of cells within depolarized (M2) and polarized (M1) mitochondrial populations. ES–Cu served as a positive control for mitochondrial depolarization. Data are presented as mean ± SD from three independent replicates.

#### Membrane integrity and viability

NCI-H460 cells (1.18 × 10^5^/well) were seeded in 12-well plates, stabilized for 24 h, and pretreated for 1 h with Fer-1, TTM, or their combination before exposure to PEG-FlpC (IC₅₀) or empty liposomes for 24 h. After treatment, cells were detached, centrifuged, and resuspended in PBS. PI (50 µg/mL) was added for 10 min at room temperature, followed by FDA (0.05 µg/mL) for 5 min. Fluorescence was acquired using a Guava^®^ EasyCyte flow cytometer (Merck Millipore; 5,000 events/sample) and analyzed in FCS Express™ 7 software (De Novo Software). Results were expressed as the percentage of viable (FDA⁺/PI⁻), early-damaged (FDA⁺/PI⁺), and dead (FDA⁻/PI⁺) cells. Data are presented as mean ± SD from three independent replicates.

#### Statistical analysis

All analyses were performed in GraphPad Prism 10.1.2. Normality, independence, and homoscedasticity were confirmed (Shapiro–Wilk, Durbin–Watson, Levene, *p* > 0.05). Parametric data were analyzed by one-way ANOVA with Dunnett’s test or Tukey’s test (*p* < 0.05). RT-qPCR values were normalized to the geometric mean of *GAPDH* and *ACTB* (Vandesompele et al. 2002), and relative expression was calculated by 2^⁻ΔΔCt^ (Pfaffl 2001). For unpaired samples, two-tailed Student’s t-tests were applied to log₂-transformed data, with *p* < 0.05 and fold change ≥ 2 or ≤ 0.5 considered statistically significant.

## Results

### PEGylated FlpC exhibits enhanced cytotoxicity and induces early morphological alterations

**Fig. 1:**
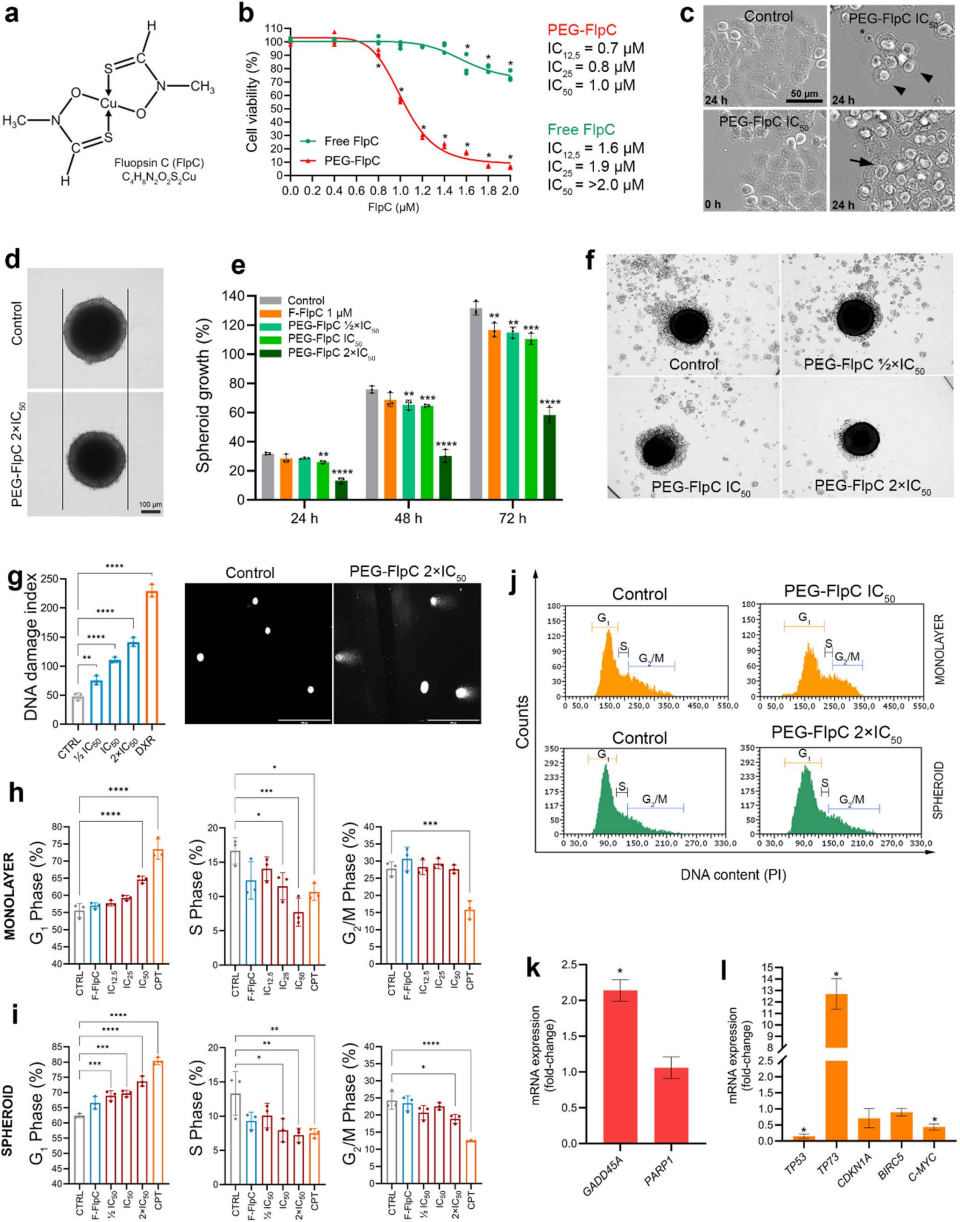
PEG-FlpC enhances cytotoxicity and induces DNA damage and G₁-phase arrest in NCI-H460 monolayers and 3D spheroids. **a** Chemical structure of FlpC. **b** Cell viability curve showing higher potency of PEG-FlpC (IC_50_ = 1.0 µM) compared to free FlpC (> 2.0 µM). **c** time-lapse images illustrating cell rounding (arrowheads), detachment, and death after PEG-FlpC (IC_50_, 24 h); arrows indicate ER-like structures; scale bar = 50 μm. **d-e** Spheroid growth inhibition by PEG-FlpC (½×, 1×, 2×IC_50_) and free FlpC (1 µM) over 72 h, with representative images **d** (scale bar = 100 μm) and quantification **e**. **f** Proliferative recovery assay after spheroid replating. Spheroids were replated after 72 h of treatment and fixed after 48 h for crystal violet staining **g** DNA damage assessed by comet assay; scale bar = 50 μm. **h-i** Cell cycle analysis in monolayer **h** and spheroids **i** showing G₁-phase arrest; Camptothecin (CPT). **j** Representative flow cytometry histograms. **k-l** RT-qPCR analysis showing upregulation of *GADD45A* **k**, and strong induction of *TP73* (12.7-fold), with concurrent downregulation of *TP53* (0.15-fold) and *C-MYC* (0.44-fold) **i**. Data are mean ± SD (*n* = 3). *p < 0.05, **p < 0.01, ***p < 0.001, ****p < 0.0001 vs. control (ANOVA, Dunnett’s test)

After 24 h of treatment, both F-FlpC (Fig. 1a) and PEG-FlpC exhibited cytotoxic effects in NCI-H460 cells (Fig. 1b; Supplementary Figs. S1 and S2). Notably, PEG-FlpC exhibited increased cytotoxic activity, reducing cell viability at lower concentrations compared to F-FlpC. The IC_12.5_, IC_25_, and IC_50_ values for PEG-FlpC were 0.75 µM, 0.88 µM, and 1.0 µM, respectively (Fig. 1b), with a dose-dependent response and a correlation of − 0.947 (R² = 0.897). For F-FlpC, the IC_12.5_ and IC_25_ values were 1.58 µM and 1.95 µM, respectively, with IC_50_ > 2.0 µM.

Guided by these cytotoxicity data, time-lapse microscopy was employed to monitor dynamic morphological changes. In the control, cells exhibited normal behavior, including division, post-division spreading and adhesion, and progressive confluence (Supplementary Video S1). In contrast, cells exposed to PEG-FlpC at IC_50_ displayed cell-cycle arrest and subsequent cell death (Supplementary Video S2). Early events included cytoplasmic alterations and the appearance of tubular structures resembling the endoplasmic reticulum, followed by loss of adhesion, cell swelling, and membrane rupture leading cell death (Fig. 1c).

### PEG-FlpC inhibits spheroid growth and impairs proliferative recovery

In control spheroids, the volume increased by 31.7%, 75.7%, and 131.6% after 24, 48, and 72 h, respectively. For F-FlpC (1 µM), the increases were 28.3%, 68.6%, and 116.6%. PEG-FlpC at ½×IC_50_, IC_50_, and 2×IC_50_ resulted in increases of 28.6%, 65.2%, and 114.8%; 25.9%, 64.5%, and 110.4%; and 13.2%, 30.0%, and 58.1%, respectively, demonstrating concentration-dependent growth inhibition (Fig. 1d-e).

Compared with the control, growth rates were significantly lower for PEG-FlpC at IC_50_ and 2×IC_50_ after 24 h, and for all concentrations after 48 and 72 h (Fig. 1e). PEG-FlpC at 2×IC_50_ produced the strongest inhibition, markedly delaying volumetric progression and showing greater antiproliferative activity than F-FlpC, which was significant only after 72 h.

To evaluate proliferative recovery, spheroids treated for 72 h were transferred to complete medium for an additional 48 h. PEG-FlpC significantly impaired recovery, particularly at 2×IC_50_, where spheroids showed minimal expansion, while control exhibited intense peripheral proliferation (Fig. 1f). These findings indicate that PEG-FlpC exerts sustained antiproliferative effects even after treatment withdrawal, suggesting prolonged therapeutic activity.

### PEG-FlpC induces DNA damage and G_1_-phase arrest in 2D and 3D models

DNA damage was assessed in 3D tumor spheroids by alkaline comet assay. Higher damage indices were observed compared with the control (damage index = 48) at all PEG-FlpC concentrations (Fig. 1g). The calculated indices were 75.7 for ½ IC_50_, 110.7 for IC_50_, and 141.7 for 2×IC_50_, indicating a dose-dependent increase in genotoxicity. In parallel, after 12 h of treatment with PEG-FlpC IC_50_, 3D spheroids exhibited a 2.2-fold upregulation of the DNA damage–responsive gene *GADD45A* (Fig. 1k), corroborating the comet assay data and confirming activation of the DNA damage response pathway.

Flow cytometry analysis revealed that PEG-FlpC induced G₁-phase arrest in both 2D monolayers and 3D spheroids (Fig. 1h-j; Supplementary Figs. S3 and S4). In 2D cultures, treatment with PEG-FlpC IC_50_ resulted in a 16.3% increase in the G₁ population compared to the control (Fig. 1h-j). In 3D spheroids, G₁ accumulation was also evident, with increases of 10.6% for ½ IC_50_, 11.8% for IC_50_, and 18.2% for 2×IC_50_ relative to the control (Fig. 1h-j).

To investigate G₁ arrest mechanisms, cell cycle–related gene expression was analyzed in 3D spheroids treated with PEG-FlpC (IC₅₀; 12 h). PEG-FlpC downregulated *TP53* (0.15-fold) and *C-MYC* (0.44-fold), while strongly upregulating *TP73* (12.7-fold) (Fig. 1l). This expression pattern suggests activation of *TP73*-mediated checkpoint control and inhibition of proliferative signaling, consistent with the observed G₁-phase arrest.

### PEG-FlpC induces ultrastructural alterations consistent with organelle stress

**Fig. 2:**
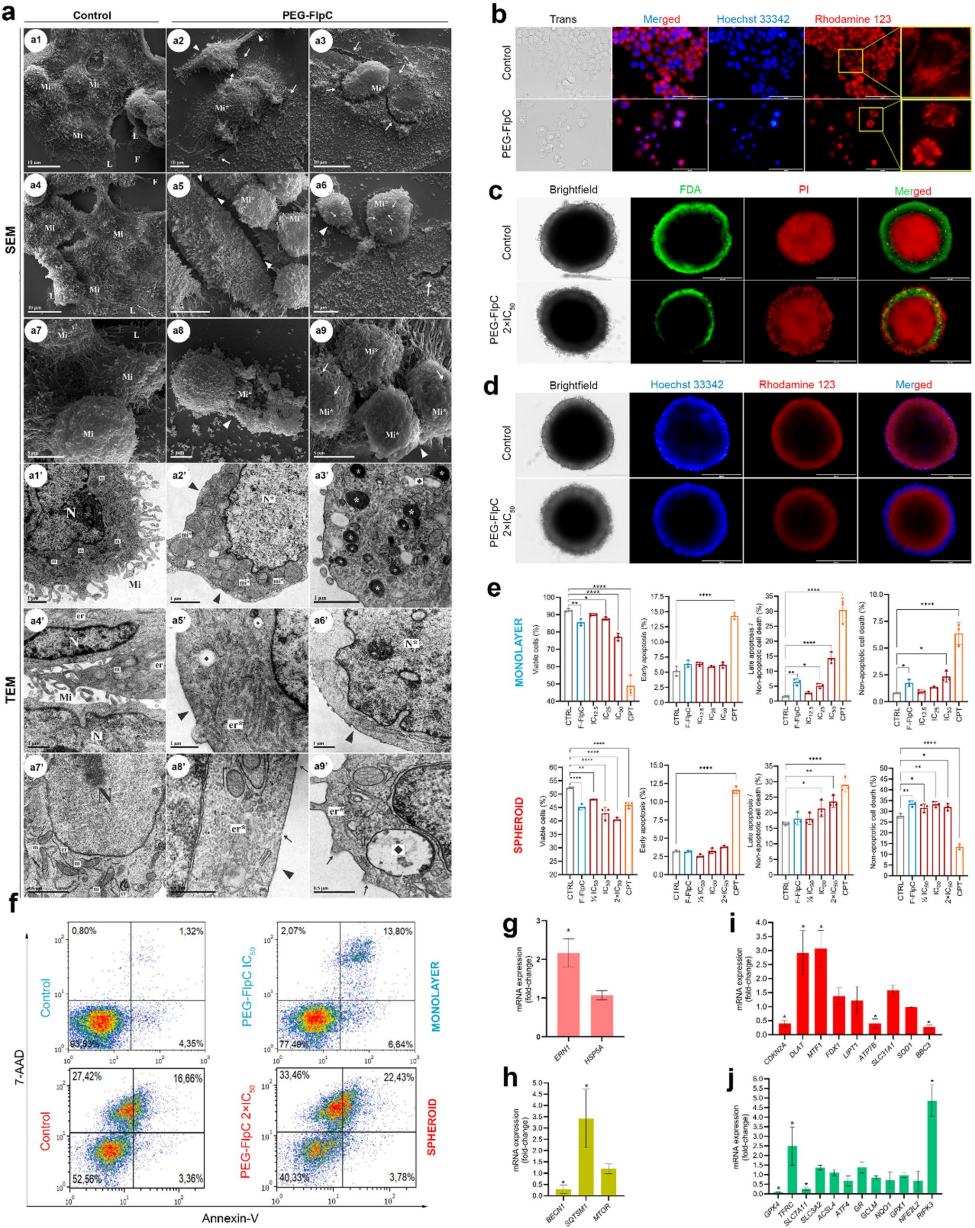
PEG-FlpC induces ultrastructural alterations, mitochondrial dysfunction, and non-apoptotic cell death in NCI-H460 monolayers and 3D spheroids. **a** scanning electron microscopy (SEM) and **a′** transmission electron microscopy (TEM) images showing morphological and ultrastructural alterations after PEG-FlpC treatment (IC_50_, 24 h). Control cells display intact membranes, abundant microvilli (Mi), lamellipodia (L), filopodia (F), and preserved mitochondria (m), endoplasmic reticulum (er), and nuclei (N). Treated cells exhibit rounding, detachment, reduced microvilli (▸), membrane rupture (→), mitochondrial swelling (mi*), ER dilation (er*), nuclear irregularities (N*), lipid droplets (*), and autophagic vacuoles (⬩). Scale bars: 10 µm (a1-a6), 5 µm (a7-a9), 1 µm (a1’-a6’), and 0.5 μm (a7’-a9’). **b** fluorescence microscopy of monolayer cells stained with Hoechst 33,342 and Rhodamine 123, showing mitochondrial depolarization and perinuclear clustering after PEG-FlpC exposure. **c-d** fluorescence microscopy of 3D spheroids stained with fluorescein diacetate (FDA) and propidium iodide (PI) **(c)** or Rhodamine 123 and Hoechst 33,342 **(d)**, revealing loss of viability and mitochondrial potential at the spheroid periphery; Scale bars: 200 μm. **e** Quantitative analysis of Annexin V/7-AAD double staining showing predominance of Annexin V⁺/7-AAD⁺ populations, consistent with non-apoptotic cell death in monolayer and spheroids. **f** Annexin V/7-AAD flow-cytometry dot plots in monolayer (top) and spheroids (bottom). **g-h** RT-qPCR showing upregulation of *ERN1* (ER stress markers, **g**) and *BECN1* and *SQSTM1* (autophagy-related genes, **h**). **i-j** modulation of cell death–related pathways: cuproptosis genes (*MTF1*, *DLAT*, *ATP7B*, and *CDKN2A*, **i**) and ferroptosis-related genes (*SLC7A11*, *GPX4*, and *TFRC*, **j**). Data represent mean ± SD (*n* = 3). *p < 0.05, **p < 0.01, ***p < 0.001, ****p < 0.0001 vs. control (one-way ANOVA, Dunnett). Camptothecin (CPT)

Ultrastructural analyses by SEM and TEM were performed after 24 h of PEG-FlpC (IC_50_) treatment (Fig. 2a). SEM showed that untreated NCI-H460 cells exhibited preserved epithelial morphology with intact membranes, abundant *microvilli*, and well-developed lamellipodia and filopodia, consistent with an active proliferative state (Fig. 2a1-a3). In contrast, PEG-FlpC-treated cells displayed rounding, loss of adhesion, reduced *microvilli*, and pronounced membrane rupture, resulting in an irregular and fragmented surface (Fig. 2a4-a9). TEM confirmed these findings, revealing mitochondrial swelling (Fig. 2a4’), cristae disorganization (Fig. 2a4’), endoplasmic reticulum dilation (Fig. 2a5’), cytoplasmic vacuolization (Fig. 2a7’), and accumulation of lipid bodies (Fig. 2a7’) and autophagic vacuoles (Fig. 2a9’). Nuclear envelope irregularities and chromatin condensation were also evident, indicating severe organelle stress and structural collapse. Consistent with these ultrastructural alterations, fluorescence microscopy revealed a perinuclear redistribution of mitochondria (Fig. 2b). Given these extensive alterations, subsequent analyses evaluated whether the observed structural changes were associated with specific cell death phenotypes.

### PEG-FlpC promotes non-apoptotic cell death in 2D and 3D tumor models

Fluorescence microscopy using FDA/PI staining revealed reduced metabolic activity in the proliferative outer layer, with increased PI uptake in the peripheral cell layer corresponding to dead cells (Fig. 2c). In parallel, Hoechst 33,342/Rhodamine 123 staining confirmed loss of mitochondrial membrane potential in peripheral cells (Fig. 2d). Consistently, flow cytometry showed a reduction in viable populations (Annexin V⁻/7-AAD⁻) following FlpC treatment (Fig. 2e-f). In 2D monolayers, the percentage of viable cells decreased by 7.5% with F-FlpC and 16.6% with PEG-FlpC at IC_50_ relative to the control. In 3D spheroids, viability decreased by 13.6% for F-FlpC, 8.3% for ½ IC_50_, 18.2% for IC_50_, and 22.4% for 2×IC_50_ (Fig. 2e-f; Supplementary Figs. S5 and S6). The proportion of Annexin V⁺/7-AAD⁺ cells (interpreted here as non-apoptotic cell death) increased in a dose-dependent manner. In 2D cultures, F-FlpC induced a 4-fold increase compared with the control, while PEG-FlpC at IC_12.5_, IC_25_, and IC_50_ induced 1.7-, 3.2-, and 9-fold increases, respectively. In 3D spheroids, the PEG-FlpC IC_50_ and 2×IC_50_ treatments resulted in 29.4% and 42.8% higher values than the control (Fig. 2e-f). When evaluating Annexin V⁻/7-AAD⁺ specifically, 2D assays showed 2.2-fold and 2.9-fold increases for F-FlpC and PEG-FlpC IC₅₀, respectively, compared with the control. In 3D spheroids, PEG-FlpC promoted increases of 20.5%, 13.5%, 19.5%, and 15.1% for F-FlpC, ½ IC₅₀, IC₅₀, and 2×IC₅₀, respectively (Fig. 2e-f).

### PEG-FlpC modulates ER stress, autophagy, and regulated cell death genes in 3D spheroids

After 12 h of PEG-FlpC exposure, the mRNA profile of 3D spheroids revealed transcriptional modulation of genes associated with endoplasmic reticulum (ER) stress, autophagy, and regulated cell death pathways (Fig. 2g-j). PEG-FlpC upregulated *ERN1* (2.2-fold), which encodes the ER stress sensor IRE1 involved in the unfolded protein response (UPR), whereas *HSPA5* showed no significant changes (Fig. 2g). Regarding autophagy, *BECN1* was downregulated (0.29-fold) and *SQSTM1* upregulated (3.4-fold), while *MTOR* expression remained unaltered (Fig. 2h). In the cuproptosis pathway, PEG-FlpC downregulated *ATP7B* (0.4-fold) and *CDKN2A* (0.4-fold), while upregulating *MTF1* (3.1-fold) and *DLAT* (2.3-fold), with no significant modulation of *FDX1*, *SLC31A1/CTR1*, *LIPT1*, or *SOD1* (Fig. 2i). In the ferroptosis pathway, *SLC7A11* (0.26-fold) and *GPX4* (0.09-fold) were downregulated, whereas *TFRC* (2.5-fold) was upregulated, with no significant changes in *SLC3A2*, *NFE2L2*, *ATF4*, *NQO1*, *ACSL4*, *GCLM*, *GSR*, or *GPX1* (Fig. 2j). Additionally, the pro-apoptotic gene *BBC3* (0.27-fold) was downregulated, suggesting suppression of canonical p53-mediated apoptosis. In contrast, the necroptosis regulator *RIPK3* (4.9-fold) was upregulated, while *RIPK1* and *MLKL* remained unchanged. Together, these transcriptional changes suggest that PEG-FlpC simultaneously engages cuproptotic and ferroptotic signaling pathways. To validate these mechanisms functionally, specific pharmacological inhibitors were employed.

### Pharmacological modulation confirms cuproptosis and ferroptosis as key mechanisms of PEG-FlpC action

**Fig. 3:**
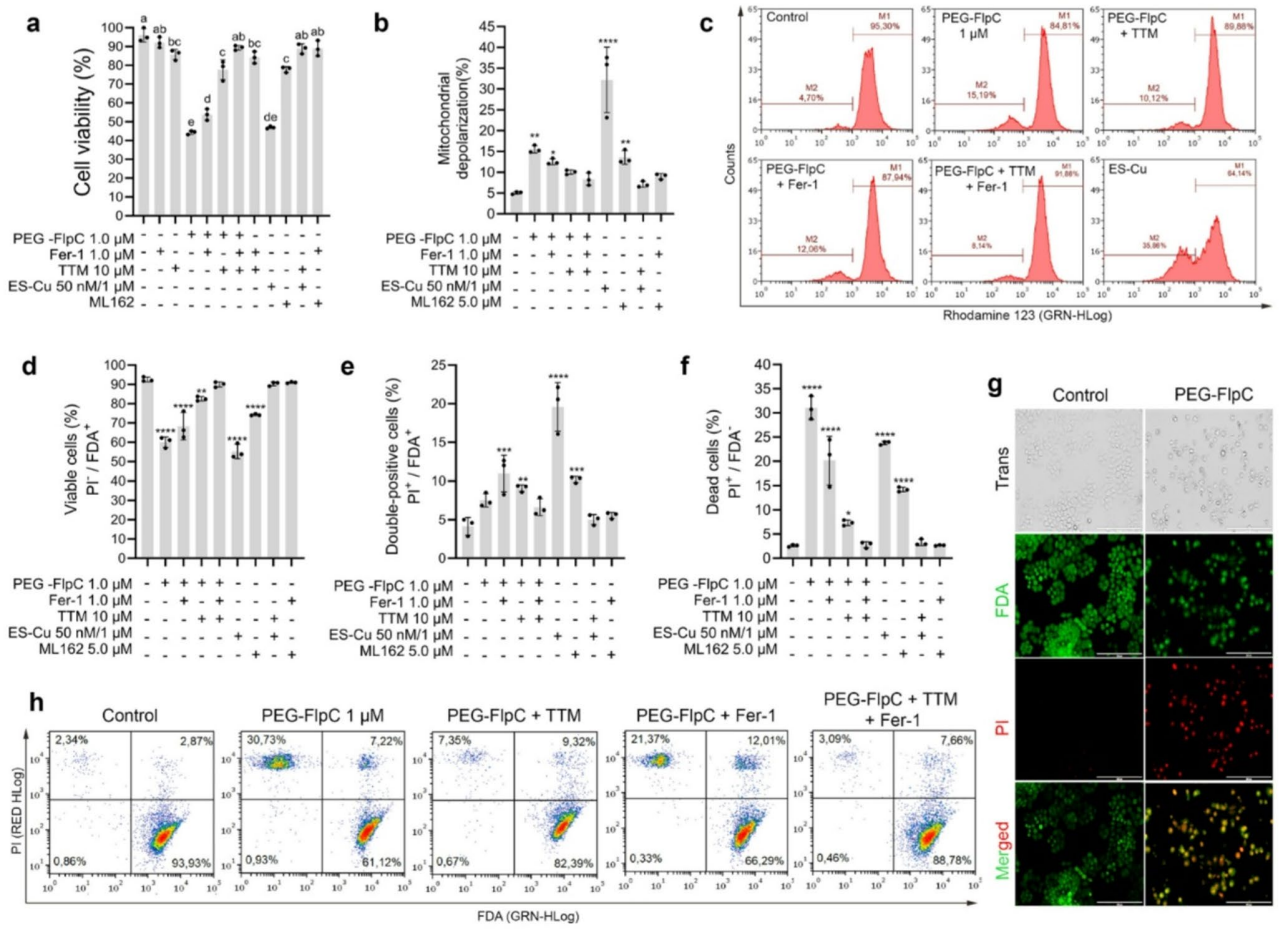
Pharmacological modulation of cuproptosis and ferroptosis attenuates PEG-FlpC-induced mitochondrial dysfunction and membrane disruption in NCI-H460 cells. **a** Cell viability determined by resazurin reduction assay showing partial recovery after pretreatment with Fer-1 (1 µM) or TTM (10 µM) and near-complete restoration with combined inhibition. **b** Quantification of mitochondrial depolarization (M2 population) assessed by Rhodamine 123 fluorescence. **c** Representative histograms illustrating mitochondrial potential (Δψmit) distribution in each treatment. **d-f** Flow-cytometric quantification of viable (FDA⁺/PI⁻), double-positive (FDA⁺/PI⁺), and necrotic (PI⁺) populations, respectively, confirming progressive membrane permeabilization following PEG-FlpC exposure and its attenuation by inhibitors. **g** Fluorescence microscopy of FDA (green) and PI (red) staining showing extensive membrane damage after PEG-FlpC. **h** Representative FDA/PI dot plots demonstrating restoration of membrane integrity with single or combined inhibition. Data are expressed as mean ± SD (*n* = 3). *p < 0.05, **p < 0.01, ***p < 0.001, ****p < 0.0001 vs. control (one-way ANOVA followed by Tukey’s test for **a** and Dunnett’s test for **b-f**). Scale bars = 200 μm

Resazurin assay showed that PEG-FlpC at IC_50_ reduced cell metabolic activity to 44.3% relative to untreated controls (Fig. 3a). Pretreatment with the ferroptosis inhibitor Fer-1 partially restored viability to 53.8%, whereas inhibition of cuproptosis with TTM produced a stronger protective effect of 77.7%. Combined treatment with both inhibitors nearly restored basal metabolic activity to 89.4%. Fer-1 and TTM alone did not alter basal metabolism, confirming the absence of intrinsic toxicity. As expected, the positive controls ML162 and Elesclomol + CuSO₄ markedly reduced metabolic activity, validating the assay conditions and confirming pathway engagement.

### Cuproptosis and ferroptosis inhibitors attenuate PEG-FlpC-induced mitochondrial depolarization

Flow cytometric analysis of Rhodamine 123 fluorescence revealed marked alterations in mitochondrial polarization following PEG-FlpC treatment. Relative to the control, PEG-FlpC increased the depolarized mitochondrial population (M2) by 3.1-fold, indicating pronounced mitochondrial dysfunction (Fig. 3b-c; Supplementary Fig. 7). Pretreatment with the ferroptosis inhibitor Fer-1 partially attenuated this effect, reducing M2 to a 2.49-fold increase, whereas inhibition of cuproptosis with TTM led to a 1.99-fold increase compared with control levels. Combined pretreatment with Fer-1 and TTM abolished this effect, showing no significant difference from the control. These results confirm that both ferroptotic and cuproptotic pathways contribute to the mitochondrial depolarization induced by PEG-FlpC.

### PEG-FlpC induces loss of membrane integrity through cuproptotic and ferroptotic mechanisms

FDA/PI staining revealed a pronounced disruption of plasma membrane integrity following PEG-FlpC exposure (Fig. 3d-h; Supplementary Fig. 8). Compared with control cells (92.6% viable; 4.2% FDA⁺/PI⁺; 2.6% PI⁺), PEG-FlpC treatment reduced viability to 60.1% and increased both early-damage (FDA⁺/PI⁺, 7.5%) and necrotic (PI⁺, 31.0%) populations, indicating extensive membrane permeabilization. Ferroptosis inhibition with Fer-1 partially mitigated these effects, restoring viability to 68.4%, reducing necrotic cells to 20.2%, while the early-damage population showed an increase (11.0%). In contrast, cuproptosis inhibition with TTM provided stronger protection, elevating viability to 82.6%, decreasing PI⁺ cells to 7.3%, and FDA⁺/PI⁺ cells to 9.1%. Combined inhibition with Fer-1 and TTM nearly restored control levels (90.0% viable; 6.6% FDA⁺/PI⁺; 2.8% PI⁺; ns). These results demonstrate that PEG-FlpC-induced membrane disruption results from the synergistic contribution of ferroptotic and cuproptotic mechanisms, with copper chelation (TTM) exerting the predominant protective effect.

## Discussion

This study demonstrates that PEG-FlpC enhances antiproliferative effects compared with the free compound, consistent with studies showing improved efficacy of PEGylated liposomal formulations over the free drug (Gu et al. 2021). Although F-FlpC shows strong antitumor activity (IC₅₀ ≤ 1.0 µM) (Ma et al. 2013; Alves de Lima et al. 2022), its application is limited by moderate hepatotoxicity and a LD₅₀ of 4 mg/kg in preclinical models (Navarro et al. 2019). In contrast, the PEGylated formulation exhibited no detectable LD₅₀ up to 8 mg/kg (Dealis Gomes et al. 2025), demonstrating improved systemic safety. The DSPE-PEG formulation was also biocompatible in vivo, causing only mild and reversible hepatic and renal alterations (Dealis Gomes et al. 2025), further supporting its translational potential for anticancer therapy.

Compared with the free compound, PEG-FlpC displayed cytotoxic and antiproliferative activity in both 2D and 3D models. F-FlpC required higher concentrations exposure, whereas PEG-FlpC induced early G₁ arrest, growth inhibition, and non-apoptotic death. In 3D spheroids, these effects led to a marked reduction in volume and proliferative recovery. These improvements are consistent with the enhanced tumor accumulation and controlled release reported for PEGylated nanocarriers (Hansen et al. 2015; Gu et al. 2021), showing that PEG-FlpC preserves and amplifies FlpC’s bioactivity while overcoming its limitations.

Ultrastructural analysis revealed mitochondrial clustering and swelling, cristae disruption, and ER dilation, features of oxidative and proteostatic stress typical of non-apoptotic regulated cell death (Dixon et al. 2012; Tsvetkov et al. 2022). PEG-FlpC-treated cells exhibited perinuclear mitochondrial aggregation and a marked reduction of surface microvilli, suggesting loss of membrane dynamics, impaired nutrient exchange, and cytoskeletal collapse. The mitochondrial depolarization and ER swelling resembled alterations caused by cuproptosis activators that aggregate lipoylated mitochondrial enzymes and promote Fe–S cluster loss (Tsvetkov et al. 2022; Jiang et al. 2024). Similar structural damage is seen in ferroptotic models, where ROS and metal redox cycling amplify oxidative stress (Miyake et al. 2020; Zhang et al. 2022). These morphological features confirm that PEG-FlpC induces organelle-centered stress.

Consistent with these structural alterations, genotoxic stress was also evident. The comet assay confirmed DNA strand breaks in spheroids, and G₁-phase accumulation was observed in both models. This is likely due to direct copper–DNA interactions causing oxidative lesions (Sangeetha and Murali 2018; Parveen et al. 2020) or to ROS generation mediated by FlpC (Ma et al. 2013). Upregulation of *GADD45A* indicates activation of DNA repair and checkpoint control (Rodríguez-Jiménez et al. 2021), consistent with our previous findings for F-FlpC in MCF-7 cells (Alves de Lima et al. 2022). Downregulation of *C-MYC* reinforces growth suppression through inhibition of proliferative signaling (Bouvard et al. 2017). Together, these results show that PEG-FlpC induces DNA damage and checkpoint activation as part of its antiproliferative effect.

Flow cytometry confirmed a reduction in viable cells and an increase in Annexin V⁺/7-AAD⁺ populations, consistent with loss of membrane integrity. The absence of nuclear fragmentation or membrane blebbing indicates activation of non-apoptotic death pathways (Sperandio et al. 2000; Green 2022). This phenotype aligns with the mitochondrial and redox disruption previously reported for copper and iron complexes (Tsvetkov et al. 2022; Zhang et al. 2022) and likely reflects effective penetration and retention of PEGylated nanocarriers in 3D spheroids (Tang et al. 2017; Niora et al. 2020). In line with these observations, the reduced expression of *BECN1* suggests impaired autophagy initiation (Li et al. 2021), whereas increased *SQSTM1* indicates a compensatory attempt at selective degradation (Sánchez-Martín et al. 2019; Guo et al. 2021). This apparent autophagy failure may lead to the accumulation of dysfunctional organelles, further aggravating mitochondrial impairment and oxidative stress. Concurrently, upregulation of *ERN1*, encoding IRE1, confirms ER stress and activation of the unfolded protein response (Le Thomas et al. 2021). Collectively, these effects, consistent with previous findings for F-FlpC (Alves de Lima et al. 2022), indicate that PEG-FlpC triggers combined mitochondrial and ER dysfunction.

Transcriptionally, PEG-FlpC modulated genes controlling cell survival and death. Downregulation of *TP53* with concurrent *TP73* upregulation suggests compensatory activation of p73-mediated transcription, typical of p53-deficient tumors (Duffy et al. 2017; Zhao et al. 2022a). Reduced *BBC3* (PUMA) expression may redirect the response toward non-apoptotic mechanisms linked to mitochondrial stress (Zheng et al. 2021).

Moreover, PEG-FlpC altered the expression of genes related to ferroptosis and cuproptosis. Downregulation of *GPX4* and *SLC7A11* with increased *TFRC* supports ferroptosis activation through impaired antioxidant defense and iron uptake (Dixon et al. 2012; Akiyama et al. 2021; Qin et al. 2024). At the same time, modulation of *ATP7B*, *MTF1*, *DLAT*, and *CDKN2A* points to copper accumulation, defective efflux, and mitochondrial proteotoxic stress characteristic of cuproptosis (Tsvetkov et al. 2022; Gao et al. 2023). The relevance of these findings is particularly pronounced in lung cancer, which exhibits a hyperactive tricarboxylic acid (TCA) cycle and elevated mitochondrial respiration (Kikuchi et al. 2020). Reduced *ATP7B* suggests limited copper export, while upregulated *MTF1* indicates activation of a metal stress response (Song et al. 2023; Zhao et al. 2022b). Increased *DLAT* supports copper-induced aggregation of mitochondrial enzymes (Ni et al. 2019). Our data also suggests activation of a non-canonical ferroptotic route, evidenced by *RIPK3* upregulation without changes in *RIPK1* or *MLKL*. This pattern may involve FSP1 inhibition via RIPK3 phosphorylation, leading to GPX4-independent lipid peroxidation and intensified ferroptotic damage (Doll et al. 2019; Lai et al. 2024). Together, these data confirm that PEG-FlpC primarily drives copper-induced toxicity, with ferroptotic signaling acting as an additional oxidative component.

Functional inhibition assays reinforce this mechanism. While Fer-1 partially restored metabolic activity, TTM provided stronger protection, nearly recovering baseline viability. The superior effect of TTM confirms that PEG-FlpC cytotoxicity is mainly driven by copper dyshomeostasis and cuproptotic mechanisms. Similar responses have been reported for Elesclomol-Cu and disulfiram derivatives, where copper chelation suppresses protein aggregation and mitochondrial collapse (Chen et al. 2021; Gao et al. 2021). These results indicate that PEG-FlpC acts through interconnected pathways of copper-induced proteotoxicity, lipid peroxidation, and autophagic dysfunction.

Despite the convergent morphological, transcriptional, and pharmacological evidence supporting cuproptotic and ferroptotic involvement, this study has limitations. Direct biochemical or protein-level markers of ferroptosis (e.g., BODIPY-C11 or MDA) and direct assessment of copper-induced protein aggregation associated with cuproptosis were not performed. Thus, although the combined structural, gene expression, and functional inhibition data strongly support the proposed mechanisms, future studies including direct functional and proteomic assays would further strengthen the mechanistic framework proposed.

In summary, PEG-FlpC acts as a copper-based nanocarrier that co-activates cuproptotic and ferroptotic pathways in NSCLC cells. By disrupting mitochondrial and ER homeostasis, it induces oxidative and proteotoxic stress leading to non-apoptotic cell death. Pharmacological inhibition confirmed the dual involvement of copper- and lipid-dependent mechanisms. These findings identify PEG-FlpC as a mechanistically distinct metallodrug with sustained antiproliferative effects and highlight its potential as a model for studying metal-driven regulated cell death in cancer.

## Supplementary Information

Below is the link to the electronic supplementary material.

Supplementary Material 1

Supplementary Material 2

Supplementary Material 3

Supplementary Material 4

## Data Availability

Data will be made available on request.
