# Supplementary material for: PEGylated liposomal fluopsin C triggers cuproptosis and ferroptosis pathways and suppresses 3D tumor spheroid growth in NCI-H460 cells
Source: Arch Toxicol. 2026 Feb 27;100(5):1981–94. doi: 10.1007/s00204-026-04315-0 (PMC13086880; doi:10.1007/s00204-026-04315-0)
Supplement: Supplementary file 1 — Supplementary Material 1 [file 204_2026_4315_MOESM1_ESM.pdf]

Supplementary Information

PEGylated Liposomal Fluopsin C triggers cuproptosis and ferroptosis pathways and suppresses 3D tumor spheroid growth in NCI-H460 cells

Luan Vitor Alves de Lima*[1], Matheus Felipe da Silva[1], Liana Martins de Oliveira[1], Maria Claudia Terkelli de Assis[1]; Isabella Cristina Oliveira Carvalho[1]; Isaura Maria Fuzinatto[1], Simone Cristine Semprebon[1], Renan Vinícius de Oliveira Nocetti[2]; Danielle Lazarin-Bidoia[2], Celso Vataru Nakamura[2], Ingrid Felicidade[1], Sandra Regina Lepri[1], Phelipe Oliveira Favaron[1], Mickely Liuti Dealis[3], Luis Fernando Cabeça[4], Galdino Andrade Filho[3], Mário Sérgio Mantovani[1]

1 Laboratory of Toxicological Genetics, Department of General Biology, Center for Biological Sciences, State University of Londrina, Londrina, Paraná, Brazil.
2 Laboratory of Technological Innovation in Drug and Cosmetic Development, Department of Basic Health Sciences, Maringá State University, Maringá, Paraná, Brazil
3 Laboratory of Microbial Ecology, Department of Microbiology, Center for Biological Sciences, State University of Londrina, Londrina, Paraná, Brazil.
4 Chemistry Laboratory, Federal Technological University of Paraná, Londrina, Paraná, Brazil.

Corresponding author: luan.vitorlima@uel.br

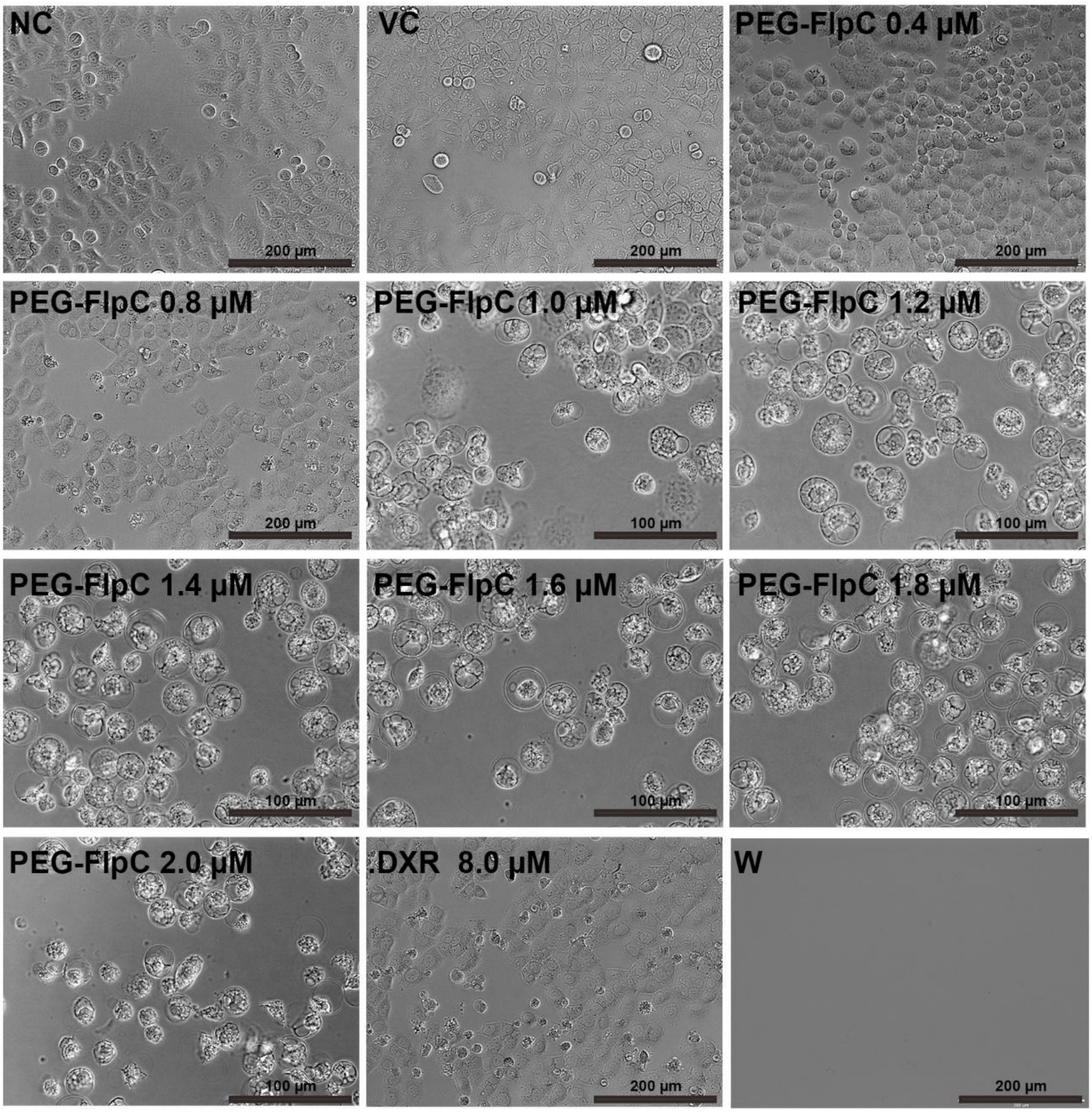

Supplementary Fig. 1 NCI-H460 human non-small cell lung carcinoma cells after 24 h of treatment with PEGylated liposomal Fluopsin C (PEG-FlpC). NC, negative control; VC, vehicle control (empty liposome); DXR, doxorubicin 8 µM; W, blank.

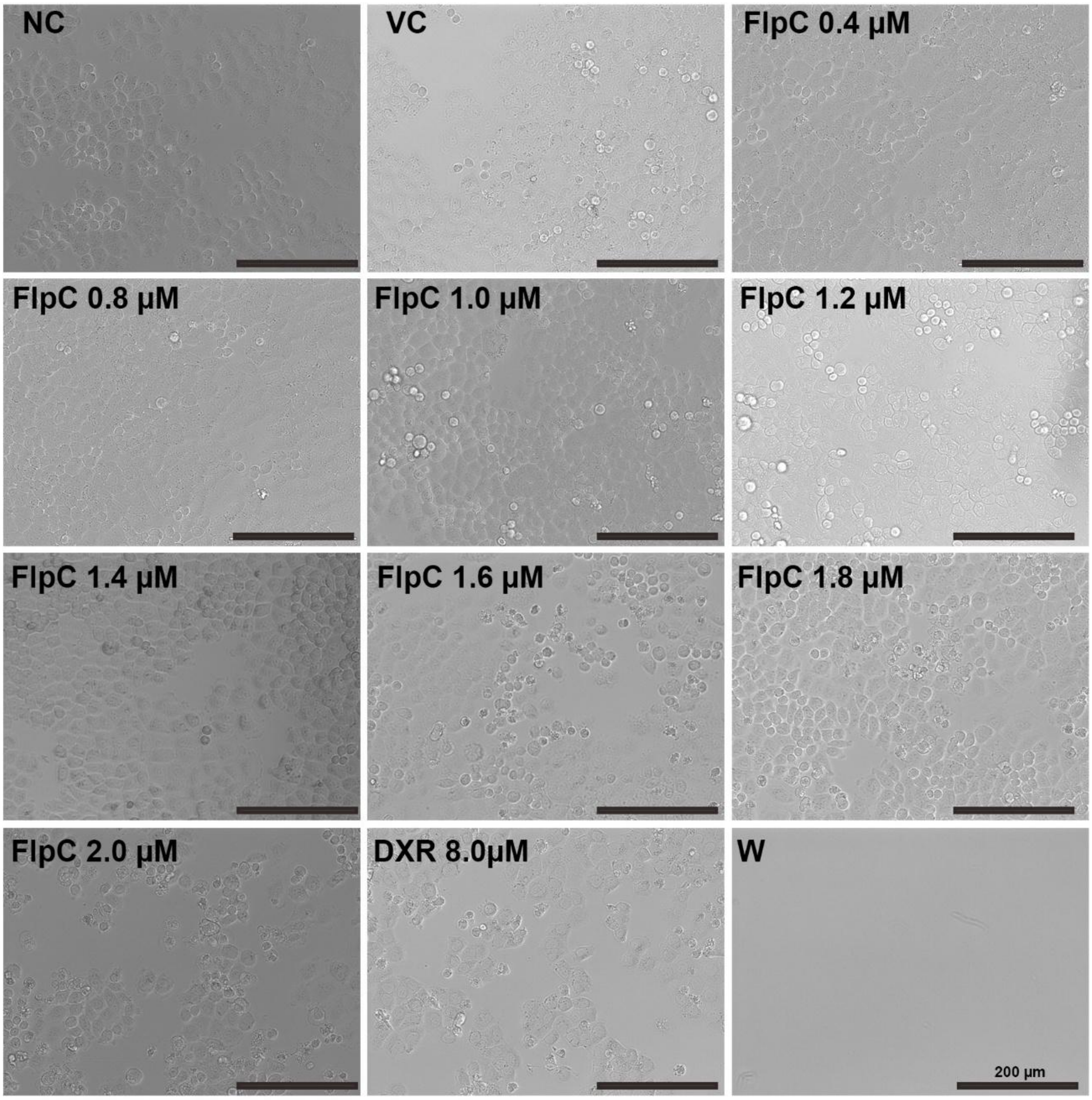

Supplementary Fig. 2 NCI-H460 human non-small cell lung carcinoma cells after 24 h of treatment with free Fluopsin C. NC, negative control; VC, vehicle control (DMSO); DXR, doxorubicin 8 μM; W, blank.

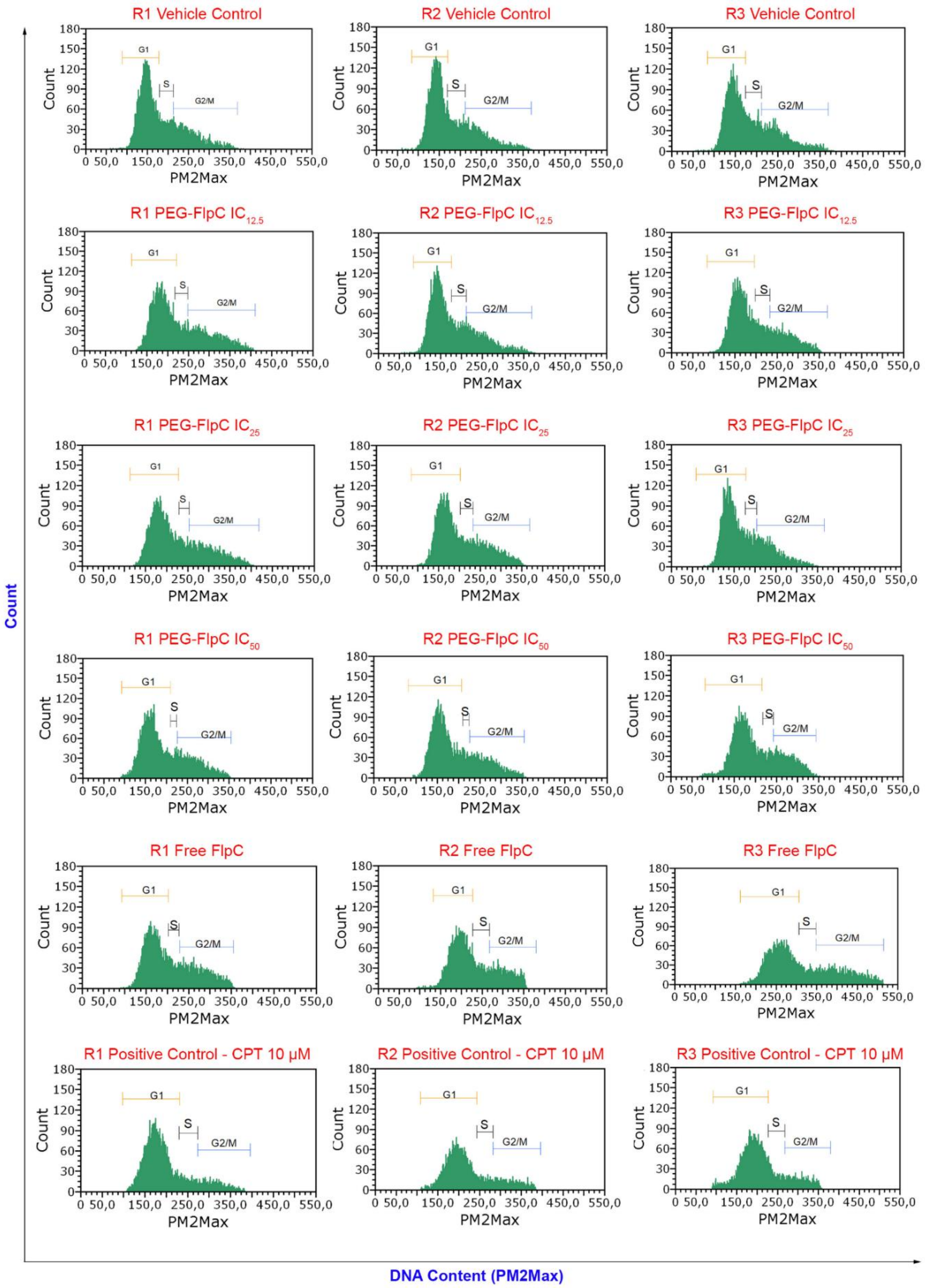

Supplementary Fig. 3 Cell cycle distribution of NCI-H460 cells after 24 h of treatment with PEGylated liposomal Fluopsin C (PEG-FlpC), free Fluopsin C (FlpC), or camptothecin (CPT, positive control). Histograms represent DNA content stained with propidium iodide (PM2Max) and acquired by flow cytometry, showing the relative proportions of cells in G_1, S, and G_2/M phases. Data are presented for three biological replicates (R1–R3) for each treatment condition.

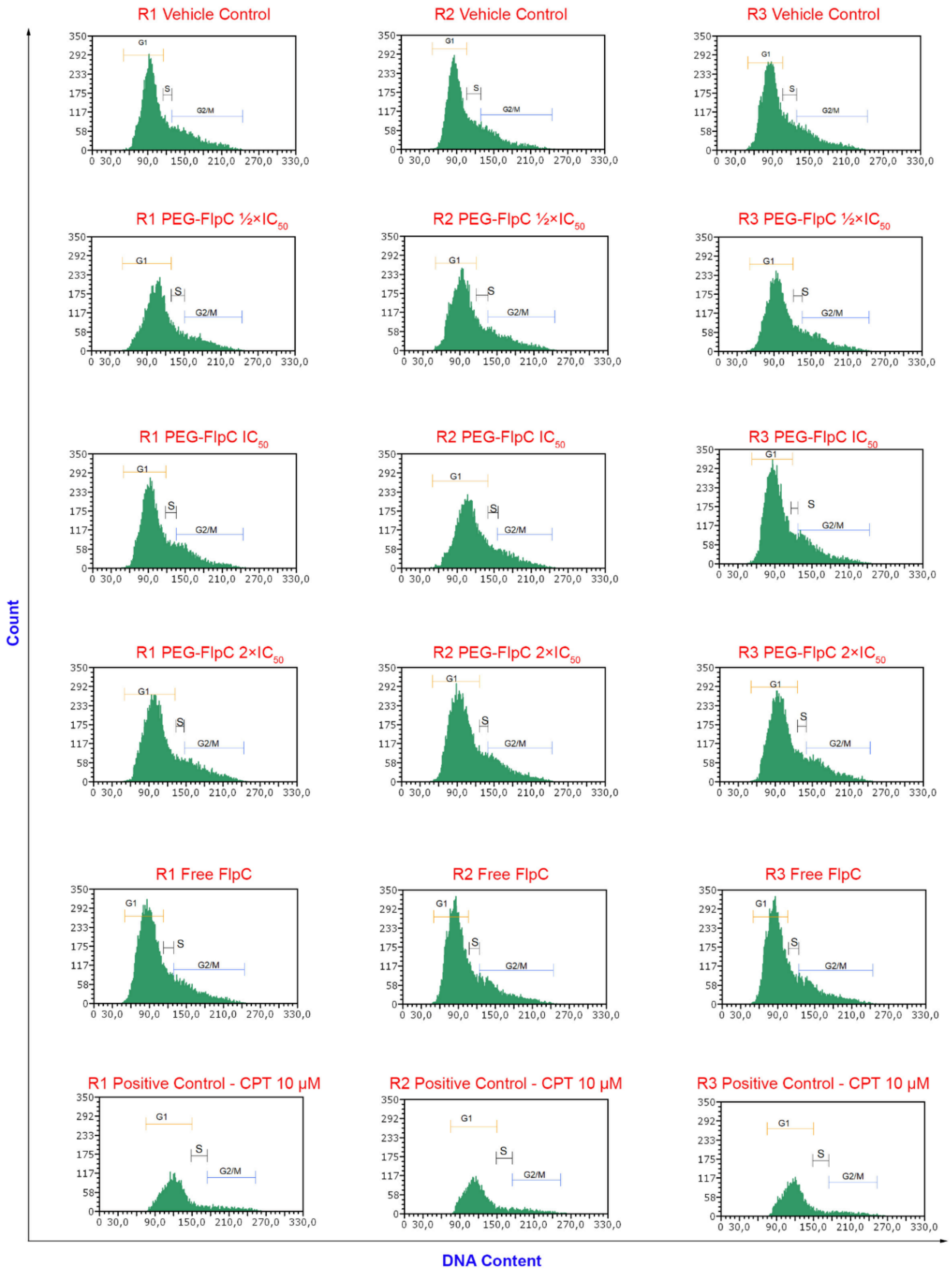

Supplementary Fig. 4 Cell cycle distribution of NCI-H460 tumor spheroids after 24 h of treatment with PEGylated liposomal Fluopsin C (PEG-FlpC), free Fluopsin C (FlpC), or camptothecin (CPT, positive control). Histograms represent DNA content stained with propidium iodide (PM2Max) and acquired by flow cytometry, showing the relative proportions of cells in G_1, S, and G_2/M phases. Each biological replicate (R1–R3) was generated from 10 spheroids.

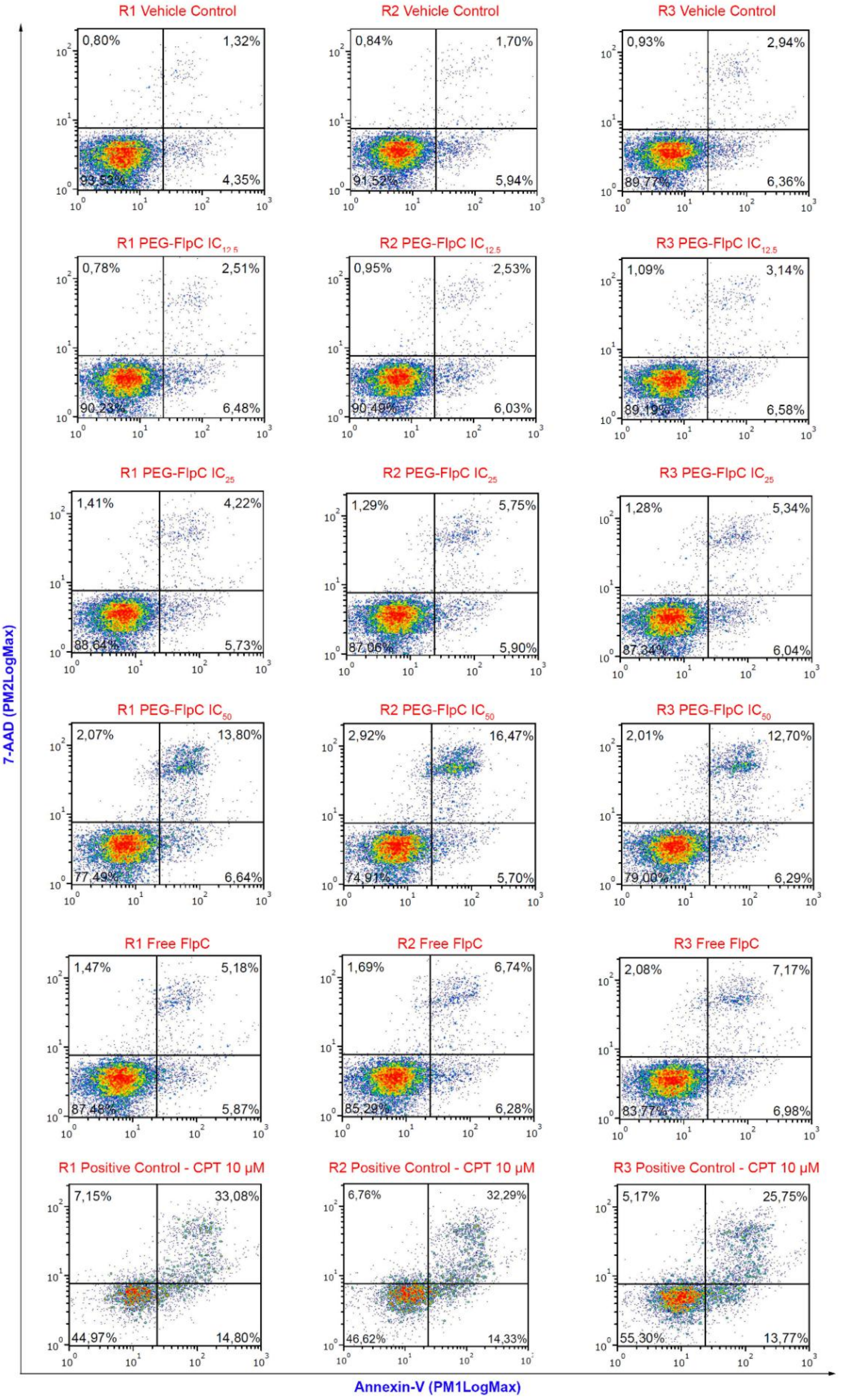

Supplementary Fig. 5 Annexin V/7-AAD staining of NCI-H460 monolayer cells after 24 h of treatment with PEGylated liposomal Fluopsin C (PEG-FlpC), free Fluopsin C (FlpC), or camptothecin (CPT, positive control). Dot plots show the distribution of viable (Annexin V$^-$/7-AAD$^-$), early apoptotic (Annexin V$^+$/7-AAD$^-$), late apoptotic (Annexin V$^+$/7-AAD$^+$), and necrotic (Annexin V$^-$/7-AAD$^+$) cells. Data are presented for three biological replicates (R1–R3) for each treatment condition.

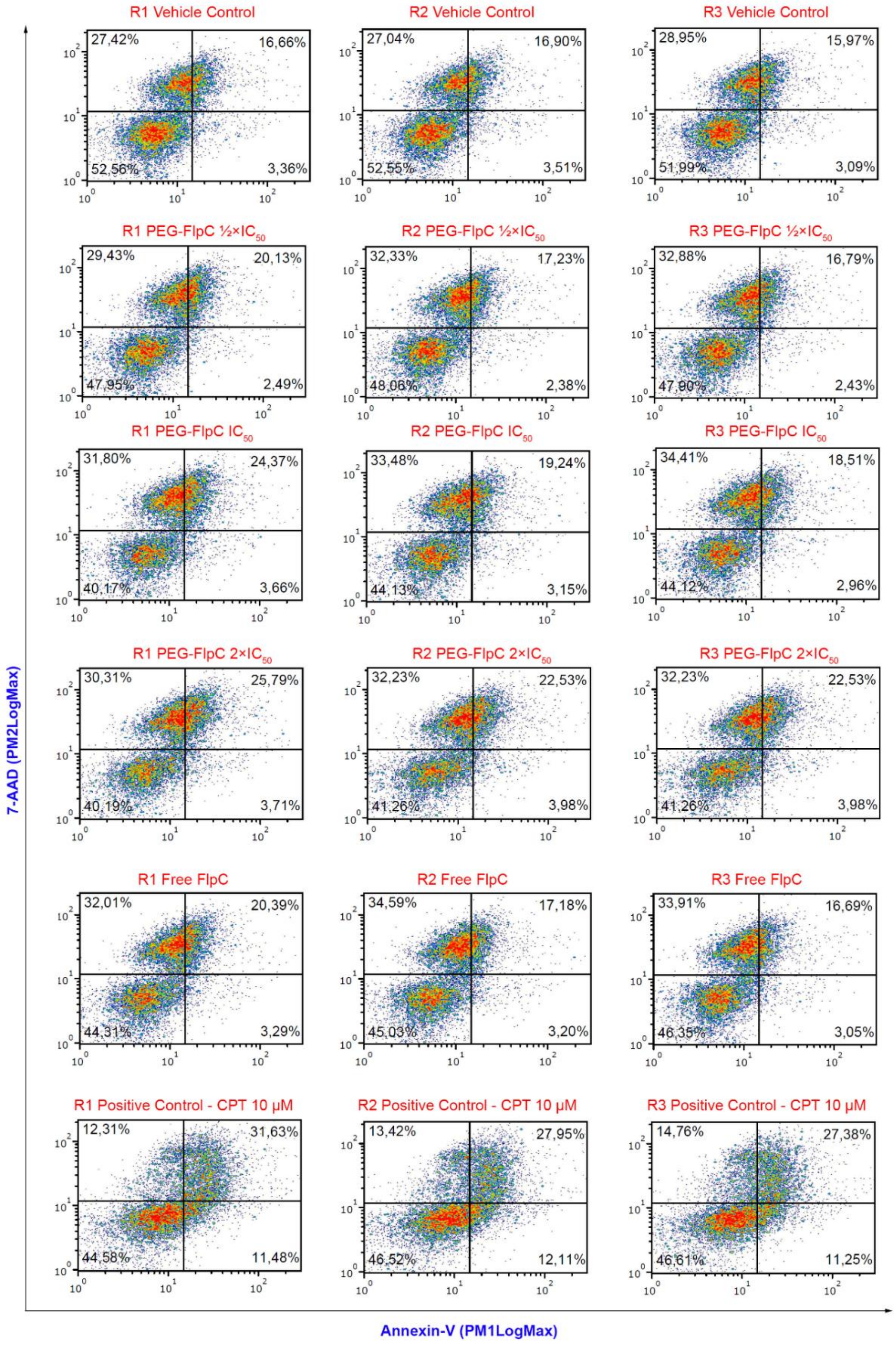

Supplementary Fig. 6 Annexin V/7-AAD staining of NCI-H460 tumor spheroids after 24 h of treatment with PEGylated liposomal Fluopsin C (PEG-FlpC), free Fluopsin C (FlpC), or camptothecin (CPT, positive control). Dot plots show the distribution of viable (Annexin V^-/7-AAD^-), early apoptotic (Annexin V^+/7-AAD^-), late apoptotic (Annexin V^+/7-AAD^+), and necrotic (Annexin V^-/7-AAD^+) cells. Each biological replicate (R1–R3) was generated from 10 spheroids.

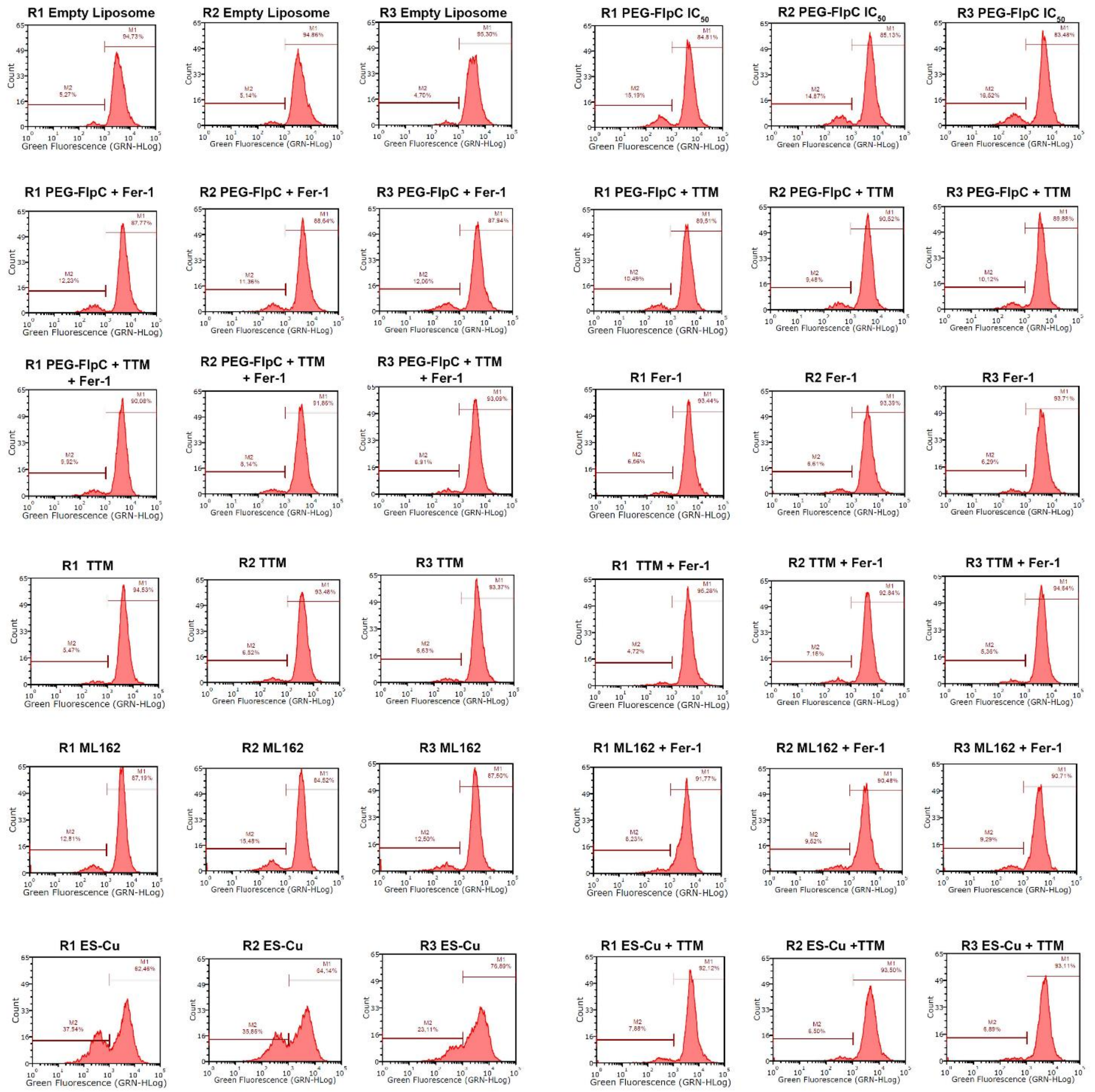

Supplementary Fig. 7 Rhodamine 123 staining of NCI-H460 cells after 24 h of treatment with PEGylated liposomal Fluopsin C (PEG-FlpC, IC_{50}), empty liposomes, or positive control ES–Cu, with or without pretreatment with Ferrostatin-1 (Fer-1), tetrathiomolybdate (TTM), or their combination. Histograms show the distribution of mitochondrial populations with preserved (M1) or depolarized (M2) membrane potential ($\Delta\psi m$), based on Rhodamine 123 fluorescence retention. Data are shown for three biological replicates (R1–R3) for each treatment condition.

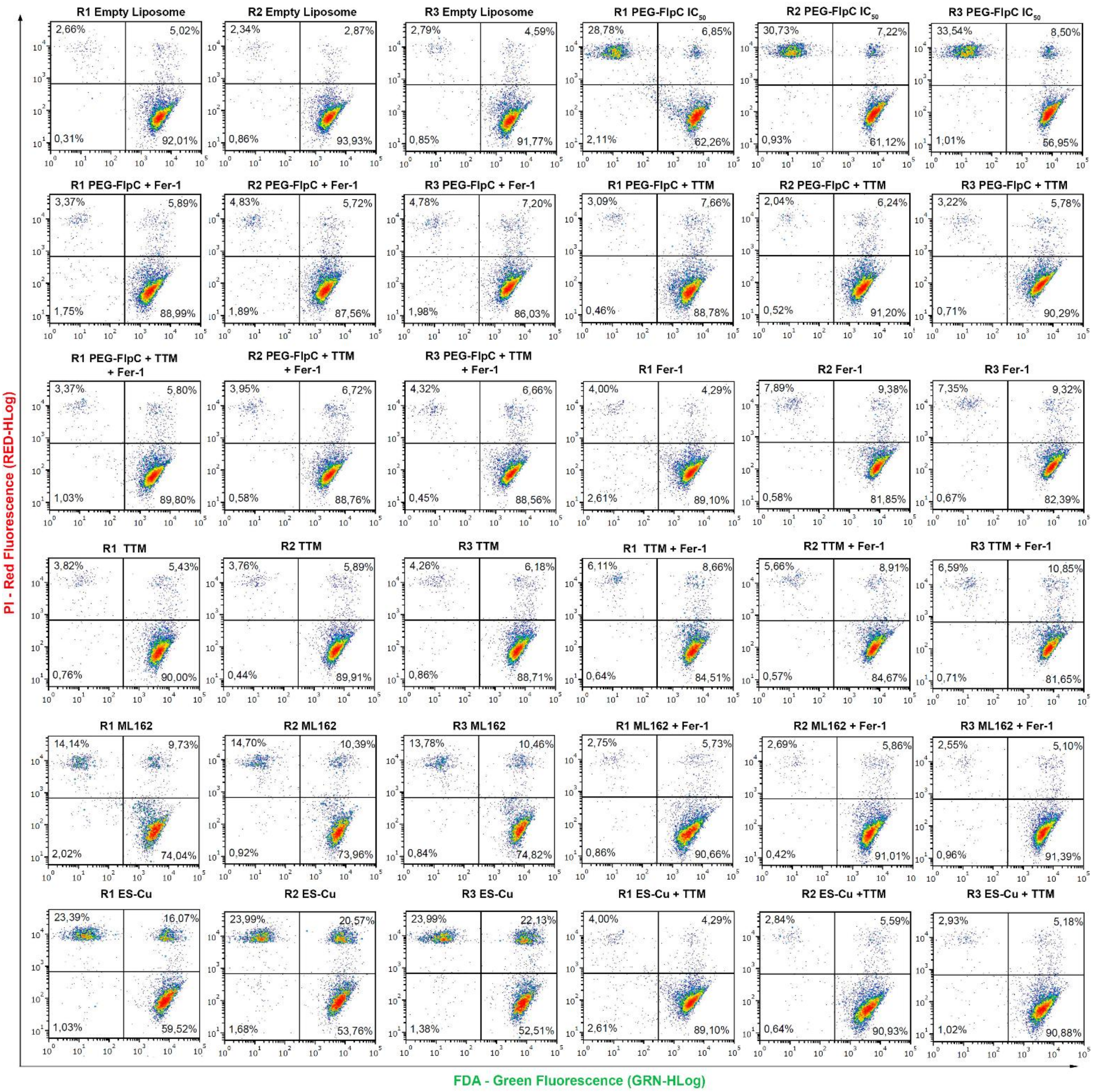

Supplementary Fig. 8 FDA/PI staining of NCI-H460 cells after 24 h of treatment with PEGylated liposomal Fluopsin C (PEG-FlpC, IC_{50}), empty liposomes, or positive controls (ML162 or ES–Cu), with or without pretreatment with Ferrostatin-1 (Fer-1), tetrathiomolybdate (TTM), or their combination. Dot plots show the distribution of viable (FDA^{+}/PI^{-}), early-damaged (FDA^{+}/PI^{+}), and dead (FDA^{-}/PI^{+}) cells based on FDA (green fluorescence) and PI (red fluorescence) uptake. Data are shown for three biological replicates (R1–R3) for each treatment condition.
