# Supplementary material for: PEGylated liposomal fluopsin C triggers cuproptosis and ferroptosis pathways and suppresses 3D tumor spheroid growth in NCI-H460 cells
Source: Arch Toxicol. 2026 Feb 27;100(5):1981–94. doi: 10.1007/s00204-026-04315-0 (PMC13086880; doi:10.1007/s00204-026-04315-0)
Supplement: Supplementary file 4 — Supplementary Material 4 [file 204_2026_4315_MOESM4_ESM.pdf]

Supplementary Information

PEGylated Liposomal Fluopsin C triggers cuproptosis and ferroptosis pathways and suppresses 3D tumor spheroid growth in NCI-H460 cells

Luan Vitor Alves de Lima*[1], Matheus Felipe da Silva[1], Liana Martins de Oliveira[1], Maria Claudia Terkelli de Assis[1]; Isabella Cristina Oliveira Carvalho[1]; Isaura Maria Fuzinatto[1], Simone Cristine Semprebon[1], Renan Vinícius de Oliveira Nocetti[2]; Danielle Lazarin-Bidoia[2], Celso Vataru Nakamura[2], Ingrid Felicidade[1], Sandra Regina Lepri[1], Phelipe Oliveira Favaron[1], Mickely Liuti Dealis[3], Luis Fernando Cabeça[4], Galdino Andrade Filho[3], Mário Sérgio Mantovani[1]

1 Laboratory of Toxicological Genetics, Department of General Biology, Center for Biological Sciences, State University of Londrina, Londrina, Paraná, Brazil.
2 Laboratory of Technological Innovation in Drug and Cosmetic Development, Department of Basic Health Sciences, Maringá State University, Maringá, Paraná, Brazil
3 Laboratory of Microbial Ecology, Department of Microbiology, Center for Biological Sciences, State University of Londrina, Londrina, Paraná, Brazil.
4 Chemistry Laboratory, Federal Technological University of Paraná, Londrina, Paraná, Brazil.

Corresponding author: luan.vitorlima@uel.br

Table S1 Cell viability (resazurin assay): Relative mean percentage of cell viability in the NCI-H460 cell line (human non-small cell lung carcinoma) after 24 h of exposure to PEGylated liposomal Fluopsin C (PEG-FlpC) and free Fluopsin C (free FlpC).

Treatment	Cell Viability (%)	
	**FlpC Formulation**	
	PEG-FlpC	***Free FplC***
NC	100	100
VC	99.76 ± 1.5	101.7 ± 0.9
0.4 μM	101.8 ± 5	101.8 ± 0.4
0.8 μM	91.87 ± 2.2 *	101 ± 2.9
1.0 μM	56.75 ± 1.8 *	97.41 ± 2.4
1.2 μM	29.78 ± 1.3 *	96.39 ± 0.4
1.4 μM	22.28 ± 1.3 *	96.14 ± 3.7
1.6 μM	17.18 ± 0.7 *	81.86 ± 7.8 *
1.8 μM	7.36 ± 0.6 *	80.44 ± 2.6 *
2.0 μM	6.37 ± 0.7 *	74.39 ± 3.8 *
PC	24.88 ± 2.2 *	28.59 ± 2.7 *

(*) $p < 0.05$ compared to the negative control, as determined by one-way analysis of variance (ANOVA) followed by Dunnett's post hoc test. Values are expressed as mean ± SD. NC, negative control (complete medium only); VC, vehicle control (empty PEGylated liposomes for PEG-FlpC; DMSO 0.0066% for free FlpC); PC, doxorubicin 8 μM).

Table S2 Forward and reverse primer sequences of the target genes used in RT–PCR.

Category	Target Genes	Company	Forward 5' → 3'	Reverse 5' → 3'
Constitutive	*ACTB*	Sigma-Aldrich	GACGACATGGAGAAAATCTG	ATGATCTGGGTCATCTTCTC
	GAPDH	Invitrogen	GAAGGTGAAGGTCGGAGTC	GGAAGATGGTGATGGGATTT
Cell Cycle Regulation	*TP53*	Sigma-Aldrich	ACCTATGGAAACTACTTCCTG	ACCATTGTTCAATATCGTCC
	TP73	Invitrogen	CATGGAGACGAGGACACGTACTAC	CTCCATCAGCTCCAGGCTCT
	C-MYC	Sigma-Aldrich	TGAGGAGGAACAAGAAGATG	ATCCAGACTCTGACCTTTTG
	CDKN1A	Sigma-Aldrich	CAGCATGACAGATTTCTACC	CAGGGTATGTACATGAGGAG
	BIRC5	Invitrogen	AGCCCTTTCTCAAGGACCAC	TGGCTCGTTCTCAGTGGGGCAGT
Apoptosis	*BBC3*	Sigma-Aldrich	GTAAGATACTGTATATGCGCTG	TTTTCCACTGTTCCAATCTG
Cuproptosis	*FDX1*	Sigma-Aldrich	AGATATTGATGGGCTTTGGTG	CAGTGATTGCATCAACTCTC
	SLC31A1	Sigma-Aldrich	TGATGCCTAGTCACCTTCTC	GAATGCTGACTTGTGACTTAC
	MTF1	Sigma-Aldrich	CACCTTTGTGTAACTCAAGG	CCTGTACAGTGTGTTGAATG
	DLAT	Sigma-Aldrich	GGTTTTGAGTACAGAGAAG	TACAATGATACAGAGTGGGG
	LIPT1	Sigma-Aldrich	GATTCTGAAGTCAACTCGC	CTGTAATCTCAGCTACTCGG
	ATP7B	Sigma-Aldrich	AAAGAGCAAACCTCAGAAG	CCCTGATGATTAAATTGTCTC
	CDKN2A	Sigma-Aldrich	AGCATGGAGCCTTCG	ATCATGACCTGGATCGG
	SOD1	Integrated DNA Technologies	CTAGCGACTTATGGCGAC	GAATGTTTATTGGGCGATC
Ferroptosis	*GPX4*	Sigma-Aldrich	GAGGTAAACTACACTCAGCTC	CTCTTTGACTCTTCGTTACTC
	GPX1	Sigma-Aldrich	CTCCCCTTACAGTGCTTGTTCG	CGATGTCAATGGTCTGGAAG
	SLC7A11	Invitrogen	TTTGCACCCTTTGACAATGA	GGAAAACAAAGCTGGGATGA
	SLC3A2	Invitrogen	CCAGGAAGGGATGATGTCGCTCAG	GAGTAAGGTCCAGAAGTGACAGG
	ACSL4	Sigma-Aldrich	ATAAAGCAGAGTACCCTGAAG	CAAGTTTTCTGGGTTAGATCC
	NFE2L2	Sigma-Aldrich	GAGAGCCCAGTCTTCATTGC	GTTTGGCTTCTGGACTTGGA
	NQO1	Invitrogen	AGCCCAGATATTGTGGCTGA	CGGAAGGGTCCTTTGTCATA
	ATF4	Invitrogen	TCAAACCTCATGGTCTCTCC	GCATGGTTTTCCAGGTCATCT
	TFRC	Sigma-Aldrich	AAGATTCAGGTCAAAGACAG	CTTACTATACGCCACATAACC
	GCLM	Invitrogen	TGTGTGATGCCACCAGATT	TTCACAATGACCGAATACCG
	GSR	Invitrogen	TTCCAGAATACCAACGTCAAAGG	GTTTTCGGCCAGCAGCTATTG
Necroptosis	*RIPK1*	Sigma-Aldrich	TGATAATACCACTAGTCTGACG	ACAGTTTTTCCAGTGCTTTC
	RIPK3	Sigma-Aldrich	AACTTTCAGAAACCAGATGC	GTTGTATATGTTAACGAGCGG
	MLKL	Sigma-Aldrich	GTGAAGAATGTGAAGACTGG	AAGATTTCATCCACAGAGGG
Endoplasmic reticulum stress	*ERN1*	Invitrogen	ACGGACGTCAAGTTTGATCC	TTGGTAGACGCAGACAGTGG
	HSPA5	Integrated DNA Technologies	GTGGAATGACCCGTCTGTG	TGCCCACCTCCAATATCAAC

Category	Target Genes	Company	Forward 5’ → 3’	Reverse 5’ → 3’
Autophagy	*SQSTM1*	Invitrogen	TCCGAGTGTGAATTTCCTG	AGCTGCCACACTCTCC
	MTOR	Sigma-Aldrich	GAAGTTTCCAGAGAAGATTCC	GTGATTCTGTAGTTGCCATC
	BECN1	Sigma-Aldrich	CAGTATCAGAGAGAATACAGTG	TGGAAGGTTGCATTAAAGAC
DNA Damage	*GADD45A*	Invitrogen	TCAGCGCACGAAGACTGTC	CCAGCAGGCACAACACCAC
	PARP1	Sigma-Aldrich	AAAAGGAGGTGGAAAAGATG	GCTAAGAACAACTCCTGAAG
